# Sperm DNA integrity is critically impacted by male age but does not influence outcomes of artificial insemination by husband in the Chinese infertile couples

**DOI:** 10.18632/aging.204058

**Published:** 2022-05-17

**Authors:** Yumei Luo, Shunhong Wu, Mimi Zhang, Hua Zhou, Jingru Yuan, Yiying Yang, Yufang Zhong, Qing Li, Xiaofang Sun, Xia Xu, Detu Zhu

**Affiliations:** 1 Department of Obstetrics and Gynecology, Key Laboratory for Major Obstetric Diseases of Guangdong Province, The Third Affiliated Hospital of Guangzhou Medical University, Guangzhou 510150, China; 2 Key Laboratory of Reproduction and Genetics of Guangdong Higher Education Institutes, The Third Affiliated Hospital of Guangzhou Medical University, Guangzhou 510150, China; 3 Guangzhou Key Laboratory for Clinical Rapid Diagnosis and Early Warning of Infectious Diseases, Kingmed School of Laboratory Medicine, Guangzhou Medical University, Guangzhou 510182, China; 4 Department of Clinical Laboratory, The Third Affiliated Hospital of Guangzhou Medical University, Guangzhou 510150, China

**Keywords:** sperm chromatin structure assay, spermatozoon DNA fragmentation index, high DNA stainability, male age, artificial insemination by husband

## Abstract

The sperm chromatin structure assay (SCSA) is crucial for assessing male fertility. However, the predictive value of the SCSA parameters, including the DNA fragment indices (DFI) and the percentages of high DNA stainability (HDS), for outcomes of artificial insemination by husband (AIH) remains controversial. This study aims to evaluate the correlations between SCSA parameters and male aging as well as other routine semen parameters, and explore their prognostic powers on AIH outcomes of the Chinese infertile couples. A total of 809 AIH cycles were retrospectively analyzed. The results showed that DFI in the age groups < 35 years were significantly lower than that in the age groups ≥ 35 years (P < 0.001). Meanwhile, there was no statistical difference in HDS between the age groups (P = 0.063). DFI and HDS are negatively correlated with most routine semen parameters (all P < 0.05). The chi-square and generalized linear model tests indicated that neither DFI nor HDS influenced the clinical pregnancy rate of AIH. In summary, this study found that aging is a critical factor leading to increased sperm DFI but not HDS. DFI and HDS are negatively correlated with most semen parameters but do not significantly influence AIH outcomes.

## INTRODUCTION

Sperm DNA is the carrier of paternal genetic materials, and its integrity is associated with sperm fertilization capacity and embryo development potential [1, 2]. The sperm DNA integrity could be gauged by DNA fragmentation index (DFI) as well as high DNA stainability (HDS) via sperm chromatin structure assay (SCSA). However, the correlation between SCSA parameters and routine sperm parameters is controversial. Some studies demonstrated that the routine sperm parameters and DNA damage are complementary, rather than strongly linked [3]. In contrast, some studies noted no clear correlation between DNA fragments and some routine semen parameters [4, 5]. Interestingly, increasing evidence indicated that male age harms sperm DNA integrity [6–8].

The value of SCSA parameters in predicting assisted reproduction treatments (ART) outcomes also remains controversial. On one hand, some studies indicated that high sperm DFI could lead to a reduced clinical pregnancy rate (CPR) of intrauterine insemination (IUI) [9]. Nevertheless, some studies suggested that the sperm DFI had no significant predictive value for ART outcomes [10, 11]. For cases with excessively high sperm DFI, doctors often have concerns about the harmful effects induced by high DFI, such as miscarriages, and suggest treatments of *in vitro* fertilization (IVF) or intracytoplasmic sperm injection (ICSI) other than IUI [12].

To address these issues, this study retrospectively analyzed the correlation between SCSA parameters and male age as well as routine sperm parameters of 809 cycles receiving artificial insemination by husband (AIH), and explore its influence and predictive power on the AIH outcomes.

## RESULTS

### Comparison of SCSA parameters among different male age groups

**Figure 1:**
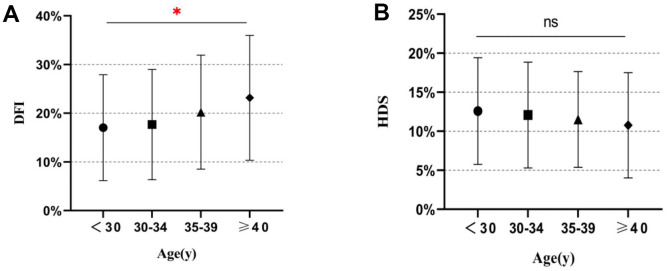
**Distribution of SCSA parameters in different age groups.** (**A**) DNA fragment index (DFI) values for different age groups. (**B**) High DNA stainability (HDS) values for different age groups. *, P < 0.05; ns, not significant.

Correlation analysis by Spearman correlation coefficients showed that the Total DFI, High DFI, and Low DFI were positively correlated with male age, while HDS has no such correlation (Table 1 and Figure 1). Furthermore, Total DFI and Low DFI in the two groups of age < 35 years were lower than those in the groups of age ≥ 35 years significantly (all P < 0.001). Besides, the High DFI of the group of age ≥ 40 years was significantly higher than those of other age groups, except the group of age 35-39 years (P < 0.001). However, regarding HDS, there was no statistically significant variation among the age groups (P = 0.063).

**Table 1:** Comparison of SCSA parameters in different male age groups.

	**<30**	**30-34**	**35-39**	**≥40**	**p-value**
Total DFI	17.08±10.88	17.71±11.33	20.27±11.72	23.34±12.85	<0.001
High DFI	5.76±7.30	6.01±5.53	6.42±4.60	7.79±5.20	<0.001
Low DFI	11.82±6.31	11.70±7.09	13.85±8.79	15.55±8.79	<0.001
HDS	12.63±6.84	12.11±6.78	11.55±6.13	10.83±6.78	0.063

Abbreviations: DFI, DNA fragmentation index; HDS, high DNA stainability; Values are presented as mean ± standard deviation.

### Correlation of SCSA parameters with the other semen parameters

Spearman correlation coefficients analysis between SCSA parameters and semen parameters showed that DFI parameters, but not HDS, were positively associated with semen volume and negatively correlated with semen pH. SCSA parameters are negatively correlated with most routine semen parameters pre- and post-processing, and sperm motility and kinetic parameters (Tables 2, 3). Regarding sperm morphology, the SCSA parameters showed positive correlations with most sperm morphological parameters, including sperm deformity index (SDI), teratozoospermia index (TZI), sperm headpiece deformity rate (H%), sperm middle piece deformity rate (M%), sperm principal piece deformity (P%), sperm abnormal form rate and sperm head area. The sperm head elongation was only relevant to High DFI (Table 4).

**Table 2:** Correlation between SCSA parameters and semen parameters pre- and post-processing.

**Variables**	**Total DFI**	**High DFI**	**Low DFI**	**HDS**
Volume	0.095**	0.082*	0.089***	NS
pH	-0.193***	-0.13***	-0.203***	NS
Pre-TMSC	-0.162***	-0.243***	-0.098**	-0.237***
Pre-concentration	-0.101**	-0.186***	NS	-0.264***
Pre-motility	-0.401***	-0.44***	-0.327***	-0.21***
Pre-PR	-0.402***	-0.432***	-0.335***	-0.188***
Post-TMSC	-0.204***	-0.275***	-0.140***	-0.222***
Post-concentration	-0.187***	-0.259***	-0.126***	-0.222***
Post-motility	-0.331***	-0.363***	-0.266***	-0.136***
Post-PR	-0.318***	-0.345***	-0.259***	-0.122***

Abbreviations: DFI, DNA fragmentation index; HDS, high DNA stainability; TMSC, total motile sperm count; PR, progressive motility. NS, not significant; * P < 0.05, ** P < 0.01, *** P < 0.001.

**Table 3:** Correlation between SCSA parameters and sperm motility parameters.

**Variables**	**Total DFI**	**High DFI**	**Low DFI**	**HDS**
ALH	-0.343***	-0.377***	-0.278***	-0.168***
VCL	-0.331***	-0.366***	-0.266***	-0.162***
VSL	-0.353***	-0.383***	-0.290***	-0.149***
VAP	-0.357***	-0.389***	-0.292***	-0.166***
LIN	-0.396***	-0.426***	-0.328***	-0.180***
STR	-0.377***	-0.408***	-0.310***	-0.179***
BCF	-0.384***	-0.433***	-0.304***	-0.225***

Abbreviations: DFI, DNA fragmentation index; HDS, high DNA stainability; TPMSC, total progressed motile sperm count; VCL, curvilinear velocity; VSL, straight-line velocity; VAP, average pathway velocity; LIN, linearity of movement; STR, straightness; BCF, beat cross frequency; ALH, amplitude of lateral head displacement. NS, not significant; * P < 0.05, ** P<0.01, *** P < 0.001.

**Table 4:** Correlation between SCSA parameters and sperm morphology parameters.

**Variables**	**Total DFI**	**High DFI**	**Low DFI**	**HDS**
SDI	0.272***	0.311***	0.209***	0.204***
TZI	0.153***	0.178***	0.117***	0.113**
Abnormal forms	0.314***	0.368***	0.234***	0.261***
H	0.304***	0.356***	0.224***	0.243***
M	0.188***	0.198***	0.154***	0.114***
P	0.230***	0.242***	0.186***	0.123***
C	NS	NS	NS	NS
sperm head area	0.224***	0.306***	0.136***	0.264***
elongation	NS	0.083*	NS	NS

Abbreviations: DFI, DNA fragmentation index; HDS, high DNA stainability; SDI, sperm deformity index; TZI, teratozoospermia index; H, sperm headpiece deformity; M, sperm middle piece deformity; P, sperm principal piece deformity; C, sperm cytoplasm deformity. NS, not significant; * P < 0.05, ** P<0.01, *** P < 0.001.

### Male age is significantly different between DFI and HDS subgroups

Also, we have applied the chi-square test to compare the clinical and demographic characteristics of the couples receiving AIH treatment among different DFI and HDS subgroups. The results showed that only male age had statistical differences between different DFI and HDS subgroups (P = 0.007 and P = 0.018, Tables 5, 6).

**Table 5:** Comparison of the clinical and demographic characteristics of the couples receiving AIH treatment among DFI and HDS subgroups.

	**DFI<30%**	**DFI≥30%**	**p**	**HDS<15%**	**HDS≥15%**	**p**
**Cycle treatment options**						
Natural cycle	246(35.24%)	39(35.14%)	0.982	221(34.99%)	64(32.82%)	0.419
Stimulated cycle	452(64.76%)	72(64.86%)		393(64.01%)	131(68.18%)	
**The number of IUI cycle**						
1	435(62.32%)	69(62.16%)	0.667	382(62.21%)	122(62.56%)	0.654
2	196(28.08%)	34(30.63%)		172(28.01%)	58(29.74%)	
≥3	67(9.60%)	8(7.21%)		60(9.77%)	15(7.69%)	
**Single/double IUI**						
single	639(91.55%)	99(89.19%)	0.415	564(91.86%)	174(89.23%)	0.259
double	59(8.45%)	12(10.81%)		50(8.14%)	21(10.77)	
**Type of infertility**						
Primary	433(62.03%)	72(64.86%)	0.567	388(63.19%)	117(60.00%)	0.423
Secondary	265(37.97%)	39(35.14%)		226(36.81%)	78(40.00%)	
**pregnancy rate**						
Pregnant	64(9.17%)	11(9.91%)	0.860	61(9.93%)	14(7.18%)	0.262
Non-pregnant	634(90.83%)	100(90.09%)		553(90.07%)	181(92.82%)	

Abbreviations: DFI, DNA fragmentation index; HDS, high DNA stainability.

**Table 6:** Comparison of the age characteristics of the couples receiving AIH treatment among DFI and HDS subgroups.

	**DFI<30%**	**DFI≥30%**	**p**	**HDS<15%**	**HDS≥15%**	**p**
**female age groups (years)**						
<30	258(36.96%)	32(28.83%)	0.129	209(34.04%)	81(41.54%)	0.156
30-34	298(42.69%)	50(45.05%)		276(44.95%)	72(36.92%)	
35-39	130(18.62%)	25(22.52%)		116(18.89%)	39(20.00%)	
≥40	12(1.72%)	4(3.60%)		13(2.12%)	3(1.54%)	
**male age groups (years)**						
<30	172(24.64%)	17(15.32%)	0.007	129(21.01%)	60(30.77%)	0.018
30-34	313(44.84)	44(39.64%)		272(44.30%)	85(43.59%)	
35-39	163(23.35%)	34(30.63%)		160(26.06%)	37(18.97%)	
≥40	50(7.16%)	16(14.41%)		53(8.63%)	13(6.67%)	

### SCSA parameters and AIH pregnancy outcomes

Furthermore, the chi-square test results showed no statistical difference between AIH clinical pregnancy rates with both DFI ≥ 30% and < 30% groups (χ2 = 0.062, P = 0.860). The AIH clinical pregnancy rate in the HDS ≥ 15% group (7.18%) was lower than that in the HDS < 15% group (9.93%), but not statistically significant (χ2 = 1.336, P = 0.262). Similarly, the generalized linear model test results showed that DFI and HDS did not influence AIH clinical pregnancy rates (Table 7).

**Table 7:** Comparison of AIH pregnancy rate among DFI and HDS subgroups.

	**Total cycle**		**Pregnant cycle**	**χ^2^**	**p-value**
DFI					
<30%	698		64 (9.17%)	0.062	0.860
≥30%	111		11 (9.91%)		
HDS					
<15%	614		61 (9.93%)	1.336	0.262
≥15%	195		14 (7.18%)		
**generalized linear model**				**Wald Chi-Squared Test**	
**Dependent variable. Pregnant/Non-pregnant**				**χ^2^**	**p-value**
DFI				0.01	0.942
HDS				1.50	0.221
DFI & HDS				0.35	0.554

Abbreviations: DFI, DNA fragmentation index; HDS, high DNA stainability.

## DISCUSSION

A strong correlation between sperm DFI/HDS and male age was shown previously. For examples, Guo et al. pointed out that aging, not routine semen parameters, was an essential influencing factor for sperm DNA integrity [13]. Das et al. reported more than twice the odds ratio for sperm DNA instability in aging males compared to young males [6]. Evenson et al. proved that the percentages of sperm HDS in males were positively correlated with age, averagely from 12.2% at age 20-25 years to 7.9% at age 60-65 years [14]. Similarly, another study indicated that men with ages > 45 years had lower sperm HDS [15]. As expected, our results showed that male aging is the leading influencing factor for increased sperm DFI, which was consistent with other reports [16–19]. And our results indicated the proportion of sperm HDS decreased with increasing age, but there was no statistical difference between men's age groups.

Recently, it was reported by Mohammadi et al. that unlike DFI, HDS was hardly associated with the classical conditions of male infertility [20]. However, some studies showed that inflammation on the male genital tract led to high DFI, which is correlated with disturbed sperm DNA integrity, which may hamper successful fertilization and induction of pregnancy [21, 22]. In addition, considerable research efforts have been devoted to revealing that both DFI and HDS were positively associated with semen volume and sperm H%, but negatively associated with sperm concentration, motility and normal form percentage [18, 23–25]. Likewise, our data exhibited that DFI and HDS were significantly associated with most semen parameters, especially sperm motility and normal form percentage, implicating sperm DNA damage as an essential cause for decreased semen quality.

The influence of DFI and HDS on AIH pregnancy outcome and its predictive value were controversial in previous studies. It was reported that men with DFI < 27% and HDS < 10% had significantly higher successful rate of pregnancy following AIH [12]. Another study suggested that DFI could serve as an independent predictor for successful pregnancy following AIH [9]. Similarly, there were reports that sperm DFI and HDS could predict men's fertility capacity, assist therapeutic decision and assess risk of congenital diseases to the newborns [14, 26]. Besides, several studies have linked DFI to miscarriage. For human fertilization and embryo development, the sperm chromatin stability is the intrinsic factor of DNA damage during fertilization pronucleus formation, in which the percentage of sperm DNA damage > 30% would likely cause male infertility [27–29]. By contrast, some scholars found that sperm DFI does not affect the AIH outcomes [10, 30]. Best et al. pointed out that neither DFI nor HDS predicted pregnancy rate as assessed by SCSA [31]. Also, a meta-analysis consisted 30 studies suggested that sperm DNA fragmentation assays could not predict ART outcomes [32]. Moreover, the American Society for Reproductive Medicine stated a lack of evidence for the association of sperm DNA damage and ART failures [33]. An interesting discovery indicated that men underwent IUI might have sperm chromatin instability, even though most of these are normozoospermic [23]. In addition, Sugihara et al. found that a therapeutic algorithm integrating sperm DNA integrity could help couples with unexplained infertility decide the treatment method and elevate the chances of successful pregnancy following IUI [34]. Our previous studies proved that cycle treatment options, single/double IUI, female age, and certain sperm parameters could predict AIH outcomes in China [35, 36]. In the current investigation, we did not identify any predictive value of sperm DFI and HDS for the AIH outcomes, which might be due to the optimized semen treatment that probably removed differences between the sperm DFI and HDS.

In summary, our study indicated that aging is a critical factor leading to increased sperm DFI in Chinese males, while it has an insignificant impact on HDS. Moreover, sperm DNA damage does cause decreased semen quality, but not significantly influence AIH outcomes in Chinese infertile couples.

## MATERIALS AND METHODS

### Patient selection

This was a retrospective cohort study enrolling patients that received AIH treatment at the fertility clinic of the Third Affiliated Hospital of Guangzhou Medical University between August 2019 and August 2020. All pregnancies were confirmed with serum positive of beta-human chorionic gonadotropin (β-hCG) on day 14 after the AIH treatment. We extracted demographic data such as the couple's age, duration of infertility, semen parameters before and after sperm processing, and the pregnancy outcomes from the patients’ records. Patients with ovarian cyst detected in the ultrasound examination, uterine lesions such as submucosal leiomyoma, and a previous diagnosis of moderate to severe pelvic endometriosis were excluded. Records with incomplete or missing data were also excluded. As a result, a total of 809 AIH cycles from 504 infertile Chinese couples were analyzed (Table 8).

**Table 8:** The clinical and demographic characteristics of the couples receiving AIH treatment among different pregnancy outcome subgroups.

	**Pregnant (n=75)**	**Non-pregnant (n=734)**	***P***
**Female age**	31.02±3.74	31.37±4.05	0.133
**Male age**	32.93±4.36	33.29±4.75	0.402
**Total DFI**	18.71±11.33	18.67±11.72	0.873
**High DFI**	6.56±5.53	6.42±8.60	0.899
**Low DFI**	12.15±7.09	12.25±6.79	0.829
**HDS**	11.76±7.06	12.01±6.61	0.715
**Volume**	2.93±1.32	2.86±1.23	0.915
**pH**	7.36±0.18	7.37±0.17	0.567
**Post-TMSC**	32.88±26.29	33.18±26.90	0.923
**Post-concentration**	70.86±53.92	71.28±55.02	0.89
**Post-motility**	93.58±7.70	93.44±7.98	0.894
**Post-PR**	87.85±12.84	89.81±10.23	0.811
**Cycle treatment options**			<0.001
Natural cycle	15(20.0%)	200(27.2%)	
Stimulated cycle	60(80.0%)	534(72.8%)	
**The number of IUI cycle**			0.049
1	42(56.1%)	399(54.2%)	
2	23(30.6%)	260(35.4%)	
≥3	10(13.3%)	75(10.2%)	
**Single/double IUI**			<0.001
single	49(65.3%)	570(77.7%)	
double	26(34.7%)	164(22.3%)	
**Type of infertility**			0.988
Primary	46(61.4%)	450(61.4%)	
Secondary	29(38.6%)	284(38.6%)	

### Semen sample collection and analysis

Semen samples were collected by masturbation on the day of ovum-pick-up or AIH. The semen specimens were kept at 37° C and were examined within half an hour after collection. After complete liquefaction, all tests were conducted according to the World Health Organization (WHO) Laboratory Manual for the Examination and Processing of Human Semen (Fifth Edition) [37]. Semen specimens were treated by density gradient centrifugation.

### Sperm chromatin structure assay

**Figure 2:**
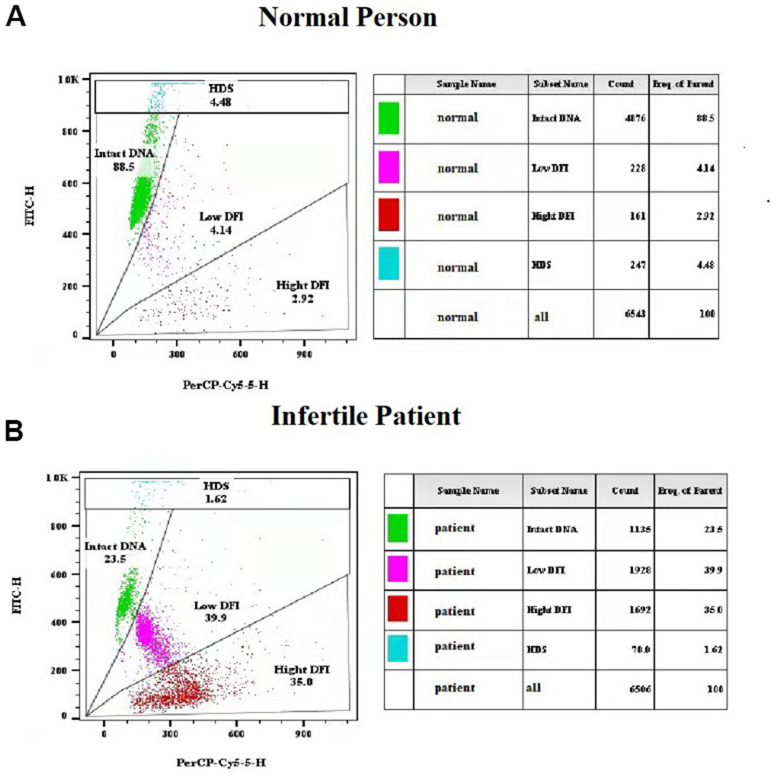
**Representative results of sperm chromatin structure assay (SCSA) using flow cytometry.** (**A**) SCSA parameters of a normal person. (**B**) SCSA parameters of an infertile patient.

Freshly liquefied semen collected after 2-7 days of abstinence were used for sperm chromatin structure assay (SCSA). All assays were completed within two months before AIH treatment following a protocol based on the previous description [38]. In brief, the sperms were stained with acridine orange solution (pH 6.0) and then analyzed with a flow cytometer (BD FACS Canto II). The sperm DNA fragment index (DFI) was calculated with the ratio of single- and double-stranded DNA, which are quantified by the red (denatured, single-stranded DNA) and green (native, double-stranded DNA) fluorescent intensities of acridine orange. DFI < 30% was defined as normal human sperm cells that contains mostly intact DNA (Figure 2A), while DFI ≥ 30% as infertile patient sperm cells that has less intact DNA and a significant number of DNA fragments based on previous reports (Figure 2B) [9, 12, 39]. Following acid exposure which caused denaturation of double-stranded DNA in immature sperms with incomplete chromatin condensation, the percentage of sperm with high DNA stainability (HDS) was quantified by the flow cytometry measurements of the metachromatic shift from green to red fluorescence.

### Intrauterine insemination

After emptying the bladder, the patient was in the bladder lithotomy position, washed the vulva with normal saline, and wiped the vagina, cervix, and fornix with a large cotton swab. A 1-mL syringe and an artificial insemination tube were connected to the uterine cavity. The catheter containing 0.5 mL of the husband’s sperm suspension is slowly placed in the uterine cavity through the cervix and about 1cm above the uterine cavity. After the semen is slowly injected into the uterus for 3 to 5 s, it is carefully withdrawn from the artificial insemination tube and speculum in the uterine cavity of the husband, and the patient is kept in the position of lowering the head and hips for about 30 min, and then can leave the operating room.

### Statistical analysis

The SPSS v22.0 was used to analyze all data. The correlations between SCSA parameters and categorical variables were evaluated by chi-square analysis. The correlations between SCSA parameters and continuous parameters were evaluated by Spearman correlation coefficients. Differences in pregnancy rates between DFI and HDS groups were evaluated by a generalized linear model. A P value < 0.05 was considered statistically significant.
